# Life Without a Breast: Exploring the Experiences of Young Nigerian Women After Mastectomy for Breast Cancer

**DOI:** 10.1200/JGO.18.00248

**Published:** 2019-05-16

**Authors:** Olalekan Olasehinde, Olujide Arije, Funmilola Olanike Wuraola, Marguerite Samson, Olawumi Olajide, Timothy Alabi, Olukayode Arowolo, Carla Boutin-Foster, Olusegun Isaac Alatise, Thomas Peter Kingham

**Affiliations:** ^1^ Obafemi Awolowo University, Ile-Ife, Osun, Nigeria; ^2^ Memorial Sloan Kettering Cancer Center, New York, NY; ^3^ State University of New York Downstate College of Medicine, Brooklyn, NY

## Abstract

**PURPOSE:**

The majority of women managed for breast cancer in Nigeria are relatively young, many in their forties. Mastectomy, the most common surgical treatment, raises psychosocial concerns. Understanding these concerns may help address the fears of women who refuse treatment and aid in the care of those who have had mastectomy.

**METHODS:**

Using qualitative methods, we purposively sampled women 45 years of age and younger who underwent mastectomy for breast cancer at a Nigerian teaching hospital. One-on-one in-depth interviews were conducted using an unstructured interview guide. Data were transcribed verbatim and analyzed to identify themes and subthemes.

**RESULTS:**

The study identified six major themes on the impact of mastectomy on psychosocial lives of women, namely decision for mastectomy, postmastectomy transition, body image changes, relationship with husband and sexual life, coping with life postmastectomy, and social support.

**CONCLUSION:**

Our findings highlight the importance of addressing individual patient’s psychosocial needs and preferences when discussing breast cancer treatment with young women. The experiences of women described in this study reveal several useful themes for planning treatment protocols and postmastectomy care.

## INTRODUCTION

Breast cancer is the most common malignancy in women and one of the leading causes of cancer death in Nigeria.^1,2^ The incidence has been projected to rise in low to middle–income countries, which is a source of concern for Nigerian communities.^3^ Equally concerning is the fact that the majority of affected women in Nigeria are relatively young.^4,5^ This background raises concerns about the quality of life of young women who have had mastectomy, either as a result of the disease or its treatment.

In Nigeria, mastectomy is the most common surgical treatment of breast cancer.^6^ Women often present with large tumors, hindering the option of breast conservation. This delay may be partly associated with the fear of mastectomy.^7^ For the majority of people, the female breast is a symbol of femininity and an important organ for child rearing.^8^ Undergoing mastectomy at a young age may therefore interfere with quality of life and overall accomplishment. These ideas may be responsible for the fear of mastectomy, which makes some women delay or refuse treatment.^7^ Aside from traditional outcomes such as recurrence and survival, there is growing interest in the quality of life of patients with breast cancer.^9-11^ Only a few studies have looked specifically at the quality of life of young women.^12^ The peculiarity of the younger patient population in the Nigerian context makes this important. Understanding the experiences of young women who have had mastectomy will help identify important aspects of care. It will also generate information that may help address fears of patients who require mastectomy at a young age. Using qualitative methods, this study explored the impact of mastectomy on the psychosocial life of women 45 years of age and younger.

## METHODS

The study was explorative, using in-depth narrative interviews to assess the psychosocial impact of mastectomy on young women who underwent mastectomy for breast cancer at a teaching hospital in South West Nigeria. The interviews were held between May 2016 and February 2017. This methodology was chosen because it does not assume a specific mind set and allows for personal narrative to evolve into general ideas and themes.

Patients were eligible to participate in the study if they were 45 years of age or younger at the time of the interview. Patients were excluded if they were older than 45 years at the time of the interview or if they had undergone breast reconstruction. Participants were identified through hospital records and contacted via telephone or recruited during clinic visits. A total of 15 interviews were conducted because this was the point at which data saturation was reached. An interview guide was developed from a review of the literature. The guide explored how interviewees felt when they were informed about mastectomy, their relationships with friends and family members, and sexual intimacy with their spouse after mastectomy. Interview questions were open ended and were not biased or leading in design. A sample of interview questions are as follows: How did you feel when you were told you will need to undergo mastectomy? Can you please explain what goes on in your mind when you look at yourself in the mirror? How have your relationships with friends and family members been since you had your mastectomy? What has been your experience with your spouse after mastectomy? What things are bothering you, which you have not had an opportunity to talk about, that you wish to tell your doctor or someone who cares to listen?

One-on-one in-depth interviews were conducted by two trained interviewers in English and in local dialect (Yoruba). Each interview lasted between 15 and 50 minutes. The interviewers participated in peer debriefing and member checking to enhance trustworthiness. All interviews were transcribed verbatim, and those conducted in local dialect were translated into English for analysis.

The analytical framework and codes were developed using ATLAS.ti 7 software. Transcripts were analyzed; recurrent, dominant, and divergent narratives were identified. Transcripts were segmented into quotes bearing meaningful concepts, which were then categorized and labeled. These were further organized into themes and subthemes on the basis of similarities and differences between contexts and phenomena. The iterative process of coding and fine tuning allowed for refinement of theoretical constructions through the linking or integration of categories around core themes. The initial coding was done by one of the researchers and fine tuning was done by a supporting researcher, both with experience in qualitative data analysis. To reduce projection and thematic leading, researchers who conducted the analyses were not involved with the interviews.

Ethical approval was obtained from Health and Research Ethics Committee of the institution (protocol number: ERC/2016/02/17, IRB/IEC/0004553), and informed consent was obtained from each participant.

## RESULTS

Fifteen women were interviewed over a 6-month period. Their ages ranged from 34 to 45 years. All women were married except one, who was separated from her spouse (Table 1). Analysis of data produced six themes.

**TABLE 1:**
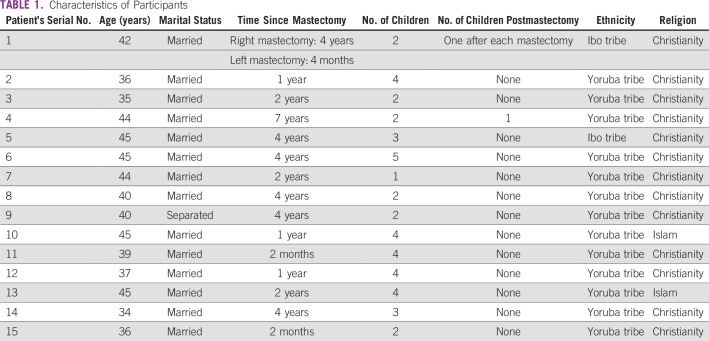
Characteristics of Participants

### Decision for Mastectomy

The decision for mastectomy was a stressor for most women. Some believed that they were faced with the choice of death or having a critical part of their body removed to save their life. A 40-year-old participant who initially refused surgery said: “I was dejected and afraid of mastectomy. Anytime they told me that I would start my treatment, I would not come as scheduled.” Testimonies from survivors of breast cancer played an important role in helping some women accept mastectomy, as depicted by a 45-year-old participant: “My doctor called one young lady who had a mastectomy. She told me she is living normally, and nobody knows unless she tells them. That was how I became encouraged.” Similarly, negative testimonies played a role, as was the case of a participant who accepted mastectomy after being told of another patient who absconded to use traditional methods and deteriorated. Some participants indicated their willingness to support others who need to undergo mastectomy, as stated by a 40-year-old participant: “I can come and encourage anybody who may need such help, you know I told you I have enjoyed from somebody.”

Having had mastectomy, some women wished they had breast-conserving operations instead, while some wished they had breast reconstruction. One participant said: “I wish I had the option of removing only the lump and not the entire breast or getting a replacement.” Another participant, who met someone who underwent breast reconstruction abroad, said: “If you looked at all of us in that gathering, you would know that we do not have a breast except for that woman who had breast reconstruction done in another country. Had we been aware, we would have opted for it.” However, one 37-year-old participant had a negative perspective toward breast reconstruction: “No, I do not like it. I know the artificial breast may cause another health problem that doctors may not be able to handle.”

### Body Image Change

Removal of the breast had a considerable impact on participants’ perceptions of femininity. A 43-year-old participant said that shortly after mastectomy, she did not like to look at herself in the mirror. Another participant reported: “I am now disabled because I am not a complete person as I am. I also know that something has gone out of me.” Another participant asked: “It is part of a woman…without a breast, are you a man?”

### Relationship With Husband and Sexual Life

For some women, informing their spouses about the need for mastectomy was the first challenge. A 39-year-old participant, when asked why she did not tell her husband until the day of the operation, said: “How would he feel if I told him? I would not even like what he would say…it would make me sorrowful.” One participant believed that spouses should be given only limited information by doctors. She said: “Maybe they should not tell them everything because I think that can discourage men.” This is quite instructive because the participant who made this comment became separated from her spouse after treatment.

Findings from this study revealed that many women experienced decreased libido after mastectomy. One participant said: “I do not have sexual urges as such. In fact, I am not interested.” The majority of women believed that breasts were foci for sexual arousal, and after mastectomy, it became difficult to become sexually aroused. One 43-year-old participant, who underwent bilateral mastectomy, explained it this way: “It is the breast that makes many women sexually aroused. It may be difficult…especially if both breasts are removed, they will need to begin to search other areas for her arousal.” Another participant said: “The particular one they removed was the one that really arouses me.” Concern about satisfying their husbands was expressed by one of the participants as follows: “How will I satisfy this man?”

### Postmastectomy Transition

The majority of women had to make lifestyle adjustments because of fear of stigmatization. One 43-year-old participant said: “Because of this operation, there was a place that we quickly constructed in my house. I told my husband, ‘you have to put a gate here; there is no way anyone will enter without knocking.’ So even if I forget to lock the front door, there is no way they will penetrate the gate and that has really helped me a lot, if not many people know.”

Some women avoided many social engagements. A 40-year-old participant said that she only attends social functions where she would be a guest and not the one to play host, which would require her to move around to serve the guests.

Another aspect of transition for women was disclosing the mastectomy to their children, many of whom were still young. One participant narrated her experience after mastectomy: “My children were looking at me but they did not pass any comment. Later, I explained what happened to the eldest one and also warned her not to tell people.” In addition, some participants had concerns about breastfeeding. Concerns border on breastfeeding in public and also on adequacy of breastfeeding a baby with only one breast. A 40-year-old participant said: “I have been wondering if this is the only breast the child will suckle and whether it will affect the baby.”

### Social Support

Participants noted that they received support from coworkers, family members, and church communities. The following quotes narrate some of these experiences.

“Since getting home, different people have been lending helping hands.” (36-year-old participant)

“I have my friends’ support and my family’s support…Since I started treatment, people have been sending money and paying my bills.” (42-year-old participant)

Some indicated that emotional support from their husbands was crucial in helping them handle their postmastectomy life. A 36-year-old participant said of her husband: “He tells me to be calm and trust in God.”

### Coping With Life Postmastectomy

Participants in this study developed certain strategies to help them cope psychologically. These include self-acceptance, distraction, lightheartedness, and religion. With respect to self-acceptance, a 35-year-old participant said*:* “Just accept yourself the way you are.” Whenever some women felt downcast, they engaged in activities that would distract them. For instance, one participant said: “At times, I would stroll out or pick up my phone.” Being lighthearted about having had the breast removed also helped some participants. One woman said: “I gave myself names like ‘mono-breasted.’” This is a form of jovial self-deprecation in which she lessened the impact of being made fun of because she could make fun of herself. Religion was an important coping strategy, as depicted by one 39-year-old participant: “The only thing that comes to my mind is praises. I always praise God for keeping me alive.” A 37-year-old participant said: “I give thanks to God…what if I was no longer alive!”

Concerning dressing, most participants hid their defects by filling their brassier with foam or strips of clothing, which many of them referred to as “packing.” The following are quotes from participants regarding this practice:

“I will just manage to pack myself well. I was told to buy foam, so I got the one that is very well packed; I will just put it double-double until it is fixed.” (43-year-old participant)

“I bought a padded bra. Once I put it on, nobody will know.” (45-year-old participant)

Another idea that emerged about coping with life after mastectomy had to do with fears of recurrence or death. A 42-year-old participant said: “I do not want recurrence. My greatest concern now is to see if anything could be done to stop the devil.” Participants also discussed their fears of the unknown or death.

## DISCUSSION

This study highlights a variety of psychosocial challenges encountered by young women who have had mastectomy for breast cancer. The impact of these challenges on their personal and family lives underscores the need to actively incorporate psychosocial rehabilitation into their care. The themes identified in this study may be useful in developing a psychosocial rehabilitation model for young women after mastectomy.

Many women who had mastectomy wished that they had breast-conserving operations or breast reconstruction instead. When compared with breast-conserving operations or mastectomy with breast reconstruction, mastectomy alone has been associated with significantly higher psychosocial morbidity in terms of body image and sexual desirability.^13-15^ Reducing the psychosocial impact of mastectomy therefore begins with measures to reduce the rate of mastectomies. Unfortunately, routine practice of breast-conserving operations is beset by various challenges peculiar to resource-limited settings such as Nigeria. First is the need for radiotherapy, which is a compulsory adjunct to breast conservation. Currently, radiotherapy centers are in short supply. The psychosocial morbidity of mastectomy on women should therefore serve as an additional motivation for improving radiotherapy services in Nigeria. Second, the majority of patients present with advanced diseases that make them ineligible for breast conservation. This makes late presentation a double jeopardy for the patient who loses her breast with poor chances of survival. Early detection therefore saves lives and also saves the breast.

Treatment providers must explore the role that women who have had mastectomies can play in assisting new patients. This study shows that their experiences can be impactful in helping women cope with the psychosocial impact of mastectomy. This can be achieved in the form of support groups where survivors share their experiences. Hearing stories of women who have had mastectomies and subsequently proceeded to have children, maintain families, and keep their jobs may help patients successfully navigate this challenging path. The role of support groups in improving the psychosocial performance of women postmastectomy has been documented.^16^ Some participants in this study indicated their willingness to participate in support groups, and this should be encouraged.

Although it is clear that women should be the focus, it is important to carefully incorporate spouses into the decision-making and rehabilitation process. A systematic review of men’s experiences of their partner’s mastectomy showed that men experienced emotional distress arising from their wives’ altered bodies, complex coping behaviors, closed communication, and consequently poor psychosocial well-being. Provision of adequate information to men was recognized as a way of overcoming these challenges.^17,18^ Provision of information to men should, however, be accompanied by adequate counseling to avoid negative consequences, as suggested by a participant who attributed her separation from her husband to the amount of information he had. Interventions involving only the woman have been considered suboptimal, whereas couple-based psychosocial interventions seem to hold better promise.^19^

Intimacy and sexual relations are aspects of patient and spousal education and counseling that must be given adequate attention. Various sexual challenges were described by participants in this study. Earlier studies, which evaluated men’s perspectives following their wives’ mastectomy, showed that sexual relationship is one of the areas where men are affected.^20^ It is therefore important to include couple counseling on sexual issues as part of the rehabilitation process. Participation in support groups by husbands and wives has also shown some benefit.^16,21^

This study shows that participants have adopted various coping measures including lightheartedness, acceptance, distraction, and religion. It also highlights the importance of social support. The religious inclination of several participants also suggests important roles for faith leaders in the rehabilitation process. These concepts can be further explored and used to develop interventional programs.

Although this study focuses on the effect of mastectomy on women’s psychosocial life, it is possible that the effect of the disease or that of other treatment modalities apart from mastectomy may have influenced their responses. The personal nature of some of the issues raised may also limit the amount of information obtained, as depicted by an interview that lasted only 15 minutes. This study may have also suffered from some recall bias, particularly by participants who had undergone mastectomy many years earlier.

In conclusion, this study highlights some important psychosocial needs and preferences of young women after mastectomy. The experiences of women described in this study are useful for planning treatment and screening protocols, as well as addressing the fears of women who are scheduled for mastectomy.
